# Conflict between Genetic and Phenotypic Differentiation: The Evolutionary History of a ‘Lost and Rediscovered’ Shorebird

**DOI:** 10.1371/journal.pone.0026995

**Published:** 2011-11-09

**Authors:** Frank E. Rheindt, Tamás Székely, Scott V. Edwards, Patricia L. M. Lee, Terry Burke, Peter R. Kennerley, David N. Bakewell, Monif Alrashidi, András Kosztolányi, Michael A. Weston, Wei-Ting Liu, Wei-Pan Lei, Yoshimitsu Shigeta, Sálim Javed, Sama Zefania, Clemens Küpper

**Affiliations:** 1 Department of Organismic and Evolutionary Biology, Harvard University, Cambridge, Massachusetts, United States of America; 2 Museum of Comparative Zoology, Harvard University, Cambridge, Massachusetts, United States of America; 3 Department of Biology and Biochemistry, University of Bath, Bath, United Kingdom; 4 Department of Biosciences, Institute of Environmental Sustainability, Swansea University, Singleton Park, Swansea, United Kingdom; 5 Department of Animal & Plant Sciences, University of Sheffield, Western Bank, Sheffield, United Kingdom; 6 Melton, Suffolk, United Kingdom; 7 Whimbrel Cottage, Wilby, Eye, Suffolk, United Kingdom; 8 Department of Biology, College of Science, University of Hail, Hail, Saudi Arabia; 9 Department of Ethology, Eötvös Loránd University, Budapest, Hungary; 10 School of Life and Environmental Sciences, Faculty of Science and Technology, Deakin University, Burwood, Victoria, Australia; 11 Taiwan Wader Study Group, Department of Environmental Science, Tunghai University, Taichung, Taiwan; 12 College of Life Science, Beijing Normal University, Beijing, China; 13 Bird Migration Research Center, Division of Avian Conservation, Yamashina Institute for Ornithology, Abiko, Chiba, Japan; 14 Biodiversity Management Sector, Environment Agency, Abu Dhabi, United Arab Emirates; 15 Department of Animal Biology, University of Antananarivo, Antananarivo, Madagascar; Biodiversity Insitute of Ontario - University of Guelph, Canada

## Abstract

Understanding and resolving conflicts between phenotypic and genetic differentiation is central to evolutionary research. While phenotypically monomorphic species may exhibit deep genetic divergences, some morphologically distinct taxa lack notable genetic differentiation. Here we conduct a molecular investigation of an enigmatic shorebird with a convoluted taxonomic history, the White-faced Plover (*Charadrius alexandrinus dealbatus*), widely regarded as a subspecies of the Kentish Plover (*C. alexandrinus*). Described as distinct in 1863, its name was consistently misapplied in subsequent decades until taxonomic clarification ensued in 2008. Using a recently proposed test of species delimitation, we reconfirm the phenotypic distinctness of *dealbatus*. We then compare three mitochondrial and seven nuclear DNA markers among 278 samples of *dealbatus* and *alexandrinus* from across their breeding range and four other closely related plovers. We fail to find any population genetic differentiation between *dealbatus* and *alexandrinus*, whereas the other species are deeply diverged at the study loci. Kentish Plovers join a small but growing list of species for which low levels of genetic differentiation are accompanied by the presence of strong phenotypic divergence, suggesting that diagnostic phenotypic characters may be encoded by few genes that are difficult to detect. Alternatively, gene expression differences may be crucial in producing different phenotypes whereas neutral differentiation may be lagging behind.

## Introduction

Explaining the occurrence and maintenance of phenotypic variation has been a central theme in evolutionary biology for more than 150 years. Darwin [1] derived many of the insights and ideas for his seminal work by observing the phenotypic diversity of pigeon breeds. However, when present in natural populations, phenotypic variation still provides challenges for taxonomy and systematics today, and the molecular machinery behind it has only just begun to be unraveled.

Phenotypic characters have been the traditional taxonomic tool of choice and continue to contribute a major share to our current understanding of the Earth's zoological diversity. Starting in the 1980s, however, a molecular revolution in the field of phylogenetics added DNA to the taxonomists' toolkit [2]. Apart from corroborating most of our long-standing classification of animals, these new molecular data routinely refine our insights into relationships between groups for which phenotypic characters seem to have been exhausted [3]. Occasionally, molecular results are at odds with previous phenotype-based hypotheses, which sometimes leads to a re-examination of the latter under more appropriate assumptions and an eventual removal of conflict [4].

Disagreement between phenotypic and molecular characters between closely related taxa can be due to inadequate data or false assumptions in at least one of the data sets, or it can be real and may point to fundamental underlying biological phenomena [5]. On the one hand, driven by molecular enquiries, there has been an unexpected abundance of discoveries of phenotypically cryptic species diversity even in such a well-studied animal clade as birds (e.g. [6], [7], [8]). Evading detection by morphological methods, cryptic species highlight the sometimes limited relevance of obvious visual cues and the vital importance of alternative signals (e.g. acoustic, chemical). On the other hand, molecular studies have yielded surprising insights into a small but growing number of species complexes in which pronounced phenotypic differences between populations or taxa are accompanied by a lack of notable sequence differentiation, including Darwin's finches, *Corvus* crows, domesticated animal breeds and humans [9], [10], [11], [12], [13], [14].

**Figure 1:**
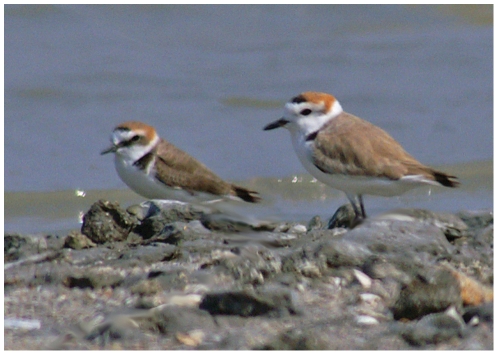
Male breeding Kentish Plover (*Charadrius alexandrinus alexandrinus*; left) and White-faced Plover (*C. a. dealbatus*; right) at Tanjung Tokong (Penang, Malaysia) by D.N. Bakewell. Note the differences in facial coloration and tone of back plumage.

When dealing with poorly-known taxa, especially those characterized by a history of shifting taxonomy, the availability of solid molecular data is vital in assessing genetic diversity and distinctness and contrasting these with phenotypic characters. Here we investigate the evolutionary history of the White-faced Plover (*Charadrius alexandrinus dealbatus*), an enigmatic East Asian shorebird that is often thought to be a subspecies of the Kentish Plover (*C. alexandrinus*). *C. alexandrinus* is a widespread breeding resident of beaches and salt pans throughout northern temperate to subtropical latitudes that has served as a model organism in ecological and evolutionary research [15], [16], [17]. Recently, Küpper *et al.* [18] restricted its range to the Old World by showing that American populations are not its sister and therefore must be considered an independent species, the Snowy Plover (*C. nivosus*). In large parts of Eurasia, there is limited morphological differentiation in *C. alexandrinus*, and most populations are considered to belong to the nominate subspecies, apart from an isolated and morphologically distinct resident population in southern India and Sri Lanka, *C. a. seebohmi*. However, in East Asia, the situation is complicated by the presence of a problematic additional taxon, *dealbatus*. Over the past few decades, most authorities (e.g. [19]) have followed Hartert & Jackson [20] in recognizing *dealbatus* as a wide-ranging but morphologically indistinct East Asian subspecies of the Kentish Plover. Only recently, field observations of unusual, distinctly pale-colored plovers in the wintering grounds of the Malayan Peninsula led to the recognition that the original description of *dealbatus* [21], [22] referred to a distinctly different plover that has been overlooked for more than a century [23]. Its name has been misapplied to birds that largely fall within the range of variation of nominate *alexandrinus* [23]. True *dealbatus* are now known to differ not only in their much paler overall plumage, but also in important details of facial coloration in breeding plumage and a range of other traits ([23]; Table 1; Fig. 1). While overlapping broadly with *alexandrinus* on migration and in the wintering grounds, *dealbatus* is only known to breed in south-east China (Fujian to Hainan provinces), to the south of the breeding distribution of *alexandrinus*, and with unknown dynamics in the area where their breeding ranges come close. Surprisingly for such a phenotypically distinct bird, *dealbatus* skins have been sitting in the drawers of major museums – unrecognized – for more than a century. Kennerley *et al.* [23] reconstruct the details of how its taxonomic identity became obliterated.

**Table 1:** Morphometric, ecological, behavioral and plumage differences between *alexandrinus* and *dealbatus* as given by Kennerley *et al.* (2008) and their interpretation and score according to the criteria of Tobias *et al.* (2010).

	*alexandrinus*	*dealbatus*	interpretation of difference (as per Tobias *et al.* 2010)	Score
plumage characters	dark lores in breeding plumage	white lores in breeding plumage	‘major’: different color of strongly demarcated body part	3
	dull dark-brown upperparts	pale brown upperparts	‘medium’: different tone of significant area of feathering	2
	narrower black frontal bar on forecrown of male breeding plumage	wider black frontal bar on forecrown of male breeding plumage	‘minor’: weak divergence in a plumage feature	1
	more dark on lower ear coverts	less dark on lower ear coverts	‘minor’, but potentially co-varying with previous traits	0
	more extensive dark patches on breast side	less extensive dark patches on breast side	‘minor’, but potentially co-varying with previous traits	0
	duller orange crown in breeding plumage	more vivid orange crown in breeding plumage	‘minor’, but potentially co-varying with previous traits	0
biometric characters	shorter wing	longer wing	effect size d = 0.448, i.e. ‘minor’	1
	shorter tarsus	longer tarsus	effect size d = 0.922, i.e. ‘minor’ (score 1), but co-varying with wing length	0
	shorter bill	longer bill	effect size d = 0.340, i.e. ‘minor’ (score 1), but co-varying with wing length	0
ecological and behavioral characters	on average inhabits softer mud along tidal channels	on average inhabits sandier substrate	‘minor’	1
	less active foraging behavior	more active foraging behavior	‘minor’ to trivial, but scoring limited to one trait	0
	horizontal stance; head held ‘hunched’ into shoulders	upright stance; neck visible	‘minor’, but scoring limited to one trait	0
geographical relationship	sympatric on migration and during winter; no information on contact of breeding ranges, therefore tentative score of 0	≥0		

Biometric measurements were taken from table 1 in Kennerley *et al.* (2008). Note that various extremely minor traits are not listed as these would not have qualified for scoring. Also note that vocal differences are not given as none are known. For the score on geographical relationship, see Results. Final score amounts to 8.

**Figure 2:**
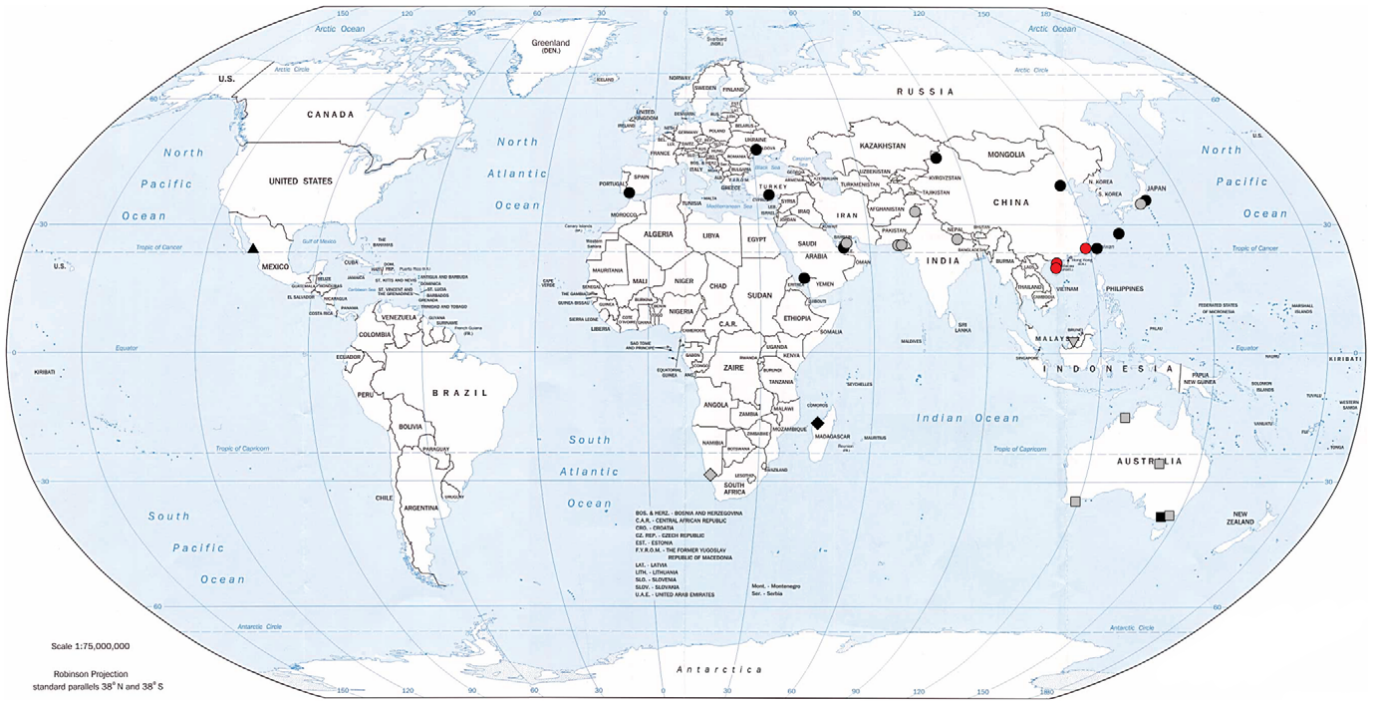
Map of collecting localities for samples. Grey symbols refer to museum specimens, while black symbols refer to blood tissue collected in the field. Red symbols refer to the three *dealbatus* localities (all of which are museum specimens). Symbol shapes refer to different species (see Table S1 in File S1 for sample sizes): upfacing triangle – *C. nivosus*; diamond – *C. marginatus*; square – *C. ruficapillus*; downfacing triangle – *C. peronii*; circle – *C. alexandrinus* (incl. *dealbatus*).

Documenting the true distribution and the pronounced phenotypic differences of a “lost” taxon of plover, Kennerley *et al.* [23] stopped short of elevating *dealbatus* to species level, instead calling for detailed molecular enquiries to examine the genetic distinctness of this enigmatic bird. Based on a large sampling regime of 278 individuals from across the globe (Fig. 2), we used three mitochondrial DNA (mtDNA) genes and seven microsatellites to investigate the evolutionary history of *dealbatus*. For a better understanding of the phylogenetic relationships of the *Charadrius alexandrinus* superspecies and to find the closest relative of *dealbatus*, we incorporated four other species that form part of the globally distributed *C. alexandrinus* complex (missing a fifth one, *C. javanicus*). Two of these (*C. ruficapillus* and *C. peronii*) as well as samples from five Asian populations of *C. alexandrinus* had not previously been available for phylogenetic research on the group [18]. To quantify phenotypic differences and further examine the biological species status of *dealbatus*, we also applied a recently proposed but promising phenotypic test of species delimitation [24], [25] to biometric, ecological, behavioural and plumage data presented by Kennerley *et al.* [23]. Given the unusually distinct breeding-plumage colouration of this “lost and found” taxon, our goal was to establish whether *dealbatus* is indeed a member of the biological species *C. alexandrinus*, and, if so, whether it exhibits any population genetic differentiation.

## Materials and Methods

### Phenotypic species delimitation

We used the species delimitation criteria recently proposed by Tobias *et al.* [24] to assess the biological species status of *dealbatus* based on the morphometric, ecological, behavioral and plumage characters presented by Kennerley *et al.* [23] (Table 1). This species delimitation test assesses phenotypic differences between two taxa by assigning each character difference a score from 1 through 4, based on whether the difference can be considered ‘minor’, ‘medium’, ‘major’ or ‘exceptional’. If the sum of scores equals or exceeds a value of seven, the magnitude of the differences suggests the separation of taxa into two distinct biological species. For exact definitions of categories and assignment rules, see Tobias *et al.* [24].

### Sampling regime and laboratory techniques

We obtained DNA from toe pad samples from museum specimens and blood samples from breeding and non-breeding populations of 278 plover individuals from across the globe (Fig. 2; Table S1 in File S1). DNA from toe pads was extracted using established protocols for museum samples at Swansea University [26]. DNA from blood samples was extracted as outlined by Küpper *et al.* [18].

For DNA samples extracted from blood we amplified three mtDNA genes: (1) a ∼400-base-pair (bp) NADH dehydrogenase subunit 3 fragment (ND3, using the L10755 and HI1151 primers from [27]), (2) a ∼1200-bp sequence including partial fragments of the ATPase subunit 6/8 genes (ATPase 6/8, using the CO2GQL and CO3HMH primers from [28]), and (3) a ∼700-bp partial D-Loop fragment of the mitochondrial control region (CR, using TS778 and SNPL90 primers from [29], [30]). All sequences are available at Genbank (accession numbers AM941499-AM941657, FR822397-FR822516, FR822850-FR822982, FR823147-FR823282), and the taxonomic identity and locality for each individual is listed in the Supplementary Material (Table S1 in File S1). For DNA obtained from toe pad samples that was partially degraded we designed primers to amplify shorter fragments. Details on these can be found in the Supplementary Material File S1. One sample from Saudi-Arabia (extracted from blood) was analysed using primers for both long and short fragments. The assembled sequences did not differ using the different primer sets and therefore we are confident that we amplified the same target region with primers for long and short fragment combinations.

MtDNA amplification conditions are given in Küpper *et al.* [18]. Initial PCRs for toe pad samples yielded little product and therefore we repeated the PCRs using 10 µg of product of the first PCR as a template. PCR products were sequenced at the NERC NBAF at the University of Edinburgh. Sequences were edited using CODONCODE ALIGNER, version 3.4 (CodonCode, Dedham, Massachusetts). Only partial sequences with both forward and reverse strands available were used in subsequent analyses. To quantify sequencing error for mitochondrial DNA from six museum skin samples, were re-extracted and then blindly re-amplified and re-sequenced.

Fragment length differences in nuclear microsatellite markers were examined in 255 individuals from twelve plover populations using six autosomal microsatellite markers developed for the Kentish Plover and one marker developed for the barn swallow (*Hirundo rustica*) (Calex–05, –11, –14, –32, –35, –37, [31]; Hru2, [32]). Each sample was run in two multiplex PCRs (MRs) containing fluorescently labelled primers (MR 1: Calex–05, –35, and Hru2 primer set; MR 2: Calex–11, –14, –32 and –37). MRs with a total volume of 10 µL contained 2–8 µL mastermix solution (Qiagen, Valencia, California), ∼2 µM of the primer mix, and 20 ng DNA. Relative primer concentrations were optimized to obtain similar peak sizes across different primer sets in the fragment analysis. MRs were performed in a thermal cycler (MJ Research model PTC DNA engine) according to the multiplex kit manufacturer's default protocol: the program started with a 15-min activation cycle at 95°C followed by 35 cycles of 94°C for 30 s, annealing temperature (MR 1: 60°C; MR 2: 62°C) for 90 s, and 90 s at 72°C. The program finished with a 10-min extension cycle at 72°C. A fraction of the MR products was loaded onto the ABI 3730, and allele sizes were assigned using GENEMAPPER, version 3.7 (Applied Biosystems, Foster City, California). Some DNA samples of museum specimens were degraded and produced inconsistent genotypes with null and/or false alleles. Therefore, for all museum samples, microsatellite genotyping was repeated four times and we only used samples that i) produced consistent genotypes across the four runs and ii) for which genotypes from at least six of the seven markers could be retrieved for the subsequent analyses.

### Phylogenetic methods

We first conducted phylogenetic analyses on individual mitochondrial loci. Subsequently, we concatenated individual loci, since all three genes are linked. The Akaike information criterion as implemented in the program jModelTest [33] was used to evaluate the best fit for each individual mtDNA gene (Table S2 in File S1). We employed maximum parsimony (MP) and Bayesian methods using the programs PAUP* 4.0b10 (Sinauer Associates, Inc.; [34]) and MRBAYES 3.1.2 [35], respectively. For details on analytical conditions of the PAUP and MRBAYES runs, see the Supplementary Material File S1.

We used a Shimodaira-Hasegawa test [36] by running 100 bootstrap replicates as implemented in PAUP to evaluate whether a tree topology constrained to *alexandrinus* and *dealbatus* monophyly had a significantly poorer fit to the sequence data than the phylogenetic tree topology obtained through Bayesian analysis. The concatenated dataset was used as input for this test, and the topology of the tree with the highest Bayesian posterior probability served as the model to compare the constrained topology against. Since separate evolutionary models cannot be specified for different data partitions of a concatenated dataset in PAUP*, the test was run three times, using each of the three evolutionary models that were found to have the best fit for each mtDNA gene, respectively.

### Population genetic methods

Basic descriptive information on the microsatellites used can be found in Küpper *et al.* [18], [31]. For microsatellite data consisting of seven loci in 180 individuals, the program ARLEQUIN version 3.11 [37] was used to compute F_ST_ values between samples unequivocally identified as *dealbatus* and *alexandrinus*. We employed the program STRUCTURAMA (http://fisher.berkeley.edu/structurama/index.html; [38]) to estimate the number of discrete populations (K) in our sampling regime. The program runs a Markov Chain Monte Carlo analysis under a Dirichlet process prior to approximate the posterior probability that individuals are assigned to specific populations. We ran two chains at 20,000,000 generations with a sampling frequency of 1,000 and excluded a burn-in of 10%.

We used STRUCTURE version 2.3.1 [39], [40] to perform ten runs of the same microsatellite dataset at K = 1 and at K = 2. Ten independent simulations were run for 1,000,000 generations with a burn-in of 100,000 using the admixture model with correlated allele frequencies. Label switching among these ten runs was taken into account and they were combined using the program CLUMPP [41] with the “FullSearch” option enforced. Structure plots were then visualized using the program DISTRUCT [42].

## Results

### Phenotypic species delimitation test

Table 1 lists biometric, ecological, behavioural and plumage differences between *alexandrinus* and *dealbatus* as given by Kennerley *et al.* [23], as well as the scores assigned to each character difference according to the phenotypic species delimitation test proposed by Tobias *et al.* [24]. The total score amounted to 8, which is greater than the “species threshold” set by Tobias *et al.* [24] at 7, indicating that *alexandrinus* and *dealbatus* display a phenotypic differentiation that is typical for members of different species.

### Sequencing and phylogenetic results

Our final alignment of overlapping CR sequences included 182 individuals and spans 580 bp, of which 116 bp were variable and 95 bp parsimony-informative. For ATPase 6/8, 168 individuals were successfully sequenced over 842 bp, of which 113 bp were variable and 90 bp parsimony-informative. Lastly, for ND3 we retrieved a 419 bp fragment for 183 individuals, 60 bp of which were variable and 45 bp parsimony-informative. The rate of sequence ambiguities for DNA samples from museum specimens was 0.3%. When concatenating our data, sequences for all three genes were available in 149 individuals spanning all the taxa examined.

**Figure 3:**
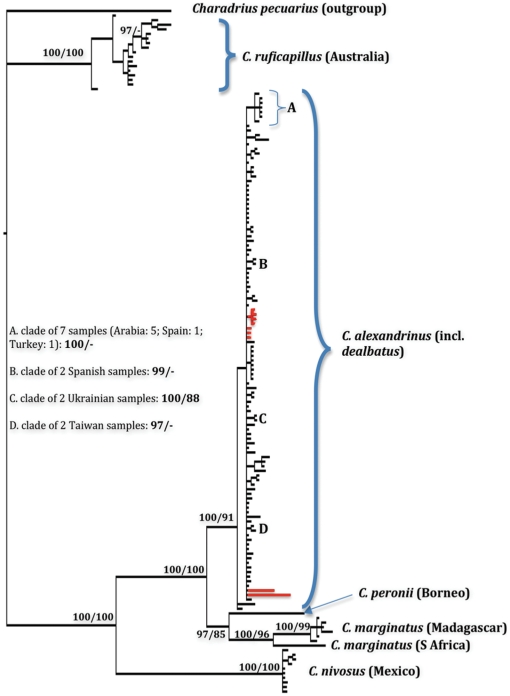
Phylogram of the concatenated dataset including all three mtDNA genes; nodal support is given in terms of Bayesian posterior probabilities (pp; multiplied by 100 for ease of interpretation) followed by maximum parsimony bootstrap (>75); only values of pp>96 are given; note that all major nodes are either highly supported (pp>96) or unsupported (pp<70), while values at 70≤pp≤96 only refer to less important population-internal nodes that are difficult to label; selected clades are letter-coded to indicate support values and sample identities; red background indicates *dealbatus* samples.

Phylogenetic results for individual loci were highly congruent to the extent that no clade strongly supported by one gene was strongly contradicted by another (data not shown). The concatenated Bayesian analysis arrived at a phylogram that corroborated the relationships uncovered by individual gene analyses and refined them with additional resolution (Fig. 3). MP analyses were usually less well-resolved than Bayesian analyses but were always in agreement.

The *dealbatus* mtDNAs were nested within a large and undifferentiated clade of *alexandrinus* mtDNAs from across Eurasia (Fig. 3), stretching from Spain in the west to Japan in the east. The combined clade of *alexandrinus* and *dealbatus* samples was characterized by high nodal support. Shimodaira-Hasegawa tests were carried out to compare maximum likelihood scores of the Bayesian concatenated topology (−ln L B) versus the score for a topology constrained to keep each *dealbatus* and *alexandrinus* monophyletic (−ln L C). Likelihood scores (−ln L B/−ln L C) were evaluated for the best-fit model of each locus (Table S2 in File S1), namely ATPase (5217.95/5628.45), ND3 (5137.72/5630.60) and CR (5204.57/5603.09). In each case, the difference between likelihoods of topologies was highly significant (p<0.005), indicating that there was no support for a tree in which *dealbatus* is monophyletic and sister to a monophyletic clade of *alexandrinus* samples.

We also recovered high nodal support for a monophyletic *C. marginatus*, although samples from mainland Africa and Madagascar are deeply diverged (Fig. 3). *C. marginatus* was placed as the sister species to *C. peronii* from south-east Asia with high support, with the clade consisting of these two species placed as the sister to *C. alexandrinus* (incl. *dealbatus*) with solid nodal support (Fig. 3). The closest relative of this lineage of three Old World plovers was the American *C. nivosus*, confirming previous results that *C. nivosus* is not the immediate sister of *C. alexandrinus* and must therefore be considered a species of its own [18]. Finally, within the limits of our sampling regime, *C. ruficapillus* from Australia was the most basal plover species within the *C. alexandrinus* superspecies, with a high support for the monophyly of the other four species (*C. alexandrinus, C. peronii, C. marginatus, C. nivosus*).

### Population genetic results

The F_ST_ value between *alexandrinus* and *dealbatus* was 0.012, indicating limited population genetic differentiation. In contrast, F_ST_ values between *dealbatus* and the three other species available for this analysis (*C. ruficapillus, C. marginatus, C. nivosus*) ranged from 0.378 to 0.405, indicating a deeper level of genetic differentiation in agreement with the results of the mtDNA analyses. In addition, F_ST_ values among those three other species were pronounced (0.478 to 0.581).

**Figure 4:**
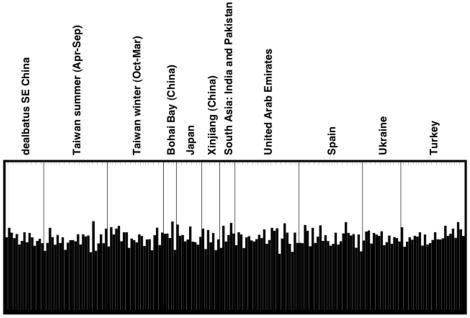
Structure plot of 176 *alexandrinus* and *dealbatus* individuals for K = 2. Note that there was no difference in the assignment probabilities between *dealbatus* and *alexandrinus* samples.

Population structure analyses of the microsatellite data of 180 *alexandrinus* and *dealbatus* specimens (not including the other plover species) using the program STRUCTURAMA yielded a probability of 0.99 for K = 1, i.e. that samples do not aggregate into multiple population clusters (with a residual probability of 0.01 for K = 2). Despite the low probability for the presence of two distinct population clusters we used STRUCTURE to compute assignment probabilities for each individual in a 2-cluster scenario (Fig. 4). Even if the presence of two population clusters was assumed, each individual (including both *dealbatus* and *alexandrinus* samples from across the entire geographic distribution) possessed a roughly equal probability of being assigned to the first versus the second cluster, indicating that there was no detectable population-genetic differentiation either between *alexandrinus* and *dealbatus*, or across Eurasian sampling sites.

## Discussion

### Phenotypic versus genetic differentiation of an enigmatic shorebird

Kennerley *et al.* [23] documented the re-discovery of a forgotten taxon of shorebird, *Charadrius alexandrinus dealbatus*, based on a suite of biometric, ecological, behavioral and plumage characters that set this form apart from nominate *alexandrinus*. While stopping short of elevating *dealbatus* to species level, the authors considered it likely that the two taxa do not interbreed and constitute distinct biological species. Our application of a phenotypic species delimitation test confirms these findings and shows that the level of phenotypic differentiation between *dealbatus* and *alexandrinus* is on a par with that of known biological species pairs.

The phenotypic species threshold at 7 is based on a calibration using a large set of known avian sister species, but Tobias *et al.* [24] acknowledge that this threshold may not be uniformly applicable and may need to be fine-tuned or adjusted in certain taxonomic groups. Our score of 8 was above the species threshold, but is probably a minimum estimate, considering our tentative assignment of a zero score to the “geographical relationship” category (Table 1): depending on the extent to which two taxa's geographic ranges come into contact, Tobias *et al.* [24] assign scores from 0 (for complete allopatry) through 3 (for parapatry), with no score assigned for sympatry since taxa automatically qualify for biological species status if they co-exist in sympatry. However, Tobias *et al.* [24] did not deal with migratory taxa such as *alexandrinus* and *dealbatus*, which are known to co-exist on migration or in the wintering grounds while keeping geographically separate on the breeding grounds. The level to which the breeding grounds of *dealbatus* and *alexandrinus* are separated is unknown: while it is unlikely that they breed sympatrically over a wide area, their breeding ranges may abut somewhere in Fujian Province (China) or they may form a narrow hybrid zone. Under these likely scenarios, a score of up to 3 could justifiably be added to the total, which would propel the test diagnostic to 11 and well beyond the gray zone around the threshold of 7. Note that vocal differences were not scored, as none are known. In summary, the application of the phenotypic species test corroborates conclusions that *dealbatus* and *alexandrinus* are phenotypically well-differentiated taxa probably worthy of species status [23].

The morphological diversification notwithstanding, *dealbatus* is characterized by little genetic differentiation from *alexandrinus* (Fig. 3, 4). In terms of mtDNA, they were virtually identical, and there were only weak differences in seven variable microsatellites. Moreover mitochondrial trees constrained to keep all *dealbatus* samples in a monophyletic group and all *alexandrinus* samples in another are significantly less likely than the undifferentiated bush-like topology for these two taxa obtained through parsimony and Bayesian analysis. The near-identical genetic signature of *dealbatus* and *alexandrinus* can be explained by three non-exclusive reasons:

1. Our genetic markers may have been too crude or too few, failing to provide sufficient population-genetic resolution. If available in sufficient quantities, nuclear population-genetic markers can discriminate between recently-differentiated populations [43], so our seven microsatellites could be argued to be insufficient to detect differentiation between *alexandrinus* and *dealbatus*. However, we discount a potential lack of resolution for two reasons: (a) For the mtDNA, the Shimodaira-Hasegawa test was not expected to show a significantly poorer fit for the monophyly-constrained topology if the undifferentiated shallow topology of the *alexandrinus-dealbatus* clade (Fig. 3) were merely due to slow sorting. Instead, if *dealbatus* and *alexandrinus* had been on a different evolutionary trajectory for a long time, there should have been an underlying signal for their separation into monophyletic groups, even if the dataset were subject to varying levels of noise by incomplete sorting. (b) The application of the same three mtDNA genes uncovers considerable differentiation among closely related plovers (Fig. 3) and other birds. By the same token, our seven microsatellite markers are unlikely to be insufficient to detect differentiation at a taxonomically recognizable level, because they showed a deep differentiation in other plover species as highlighted by large F_ST_ values (>0.35) between closely related sibling species in the *C. alexandrinus* superspecies complex. While some of our microsatellites are found in conserved genomic regions, others are located in non-conserved ones [44]. Therefore, the F_ST_ values strongly suggest that our markers were sufficient.
2. Even if they had reached species-level differentiation, *dealbatus* and *alexandrinus* may still occasionally exchange genetic material through introgression [5], especially considering that their breeding ranges may abut or narrowly overlap. Genetic introgression has been found in many birds (e.g. [45], [46], [47]) and mammals, including hominids [48]. However, while it is unknown how pervasive genetic introgression can be, present data on cases with genomic information indicate that introgression may only affect ∼5% of the nuclear genome [48]. In our study, genetic markers came from both the nuclear and mtDNA genome, and our seven microsatellites were randomly distributed throughout the nuclear genome. Based on the genomic distribution of our markers, the simultaneous impact of introgression on all loci is unlikely.
3. The low level of differentiation between *dealbatus* and *alexandrinus* may be genuine and extend to large parts of the genome. While more extensive nuclear locus sampling would be needed to rule out that we have not overlooked substantial differentiation based on marker choice, it is likely that *dealbatus* and *alexandrinus* join a list of unusual organisms that are characterized by great differentiation in phenotype but not in genotype, such as Darwin's finches, *Corvus corone* crows, domesticated animals, certain lizards and our own species *Homo sapiens* (e.g. [9], [10], [11], [12], [13], [14], [49]).

The lack of differentiation in highly variable population genetic markers may indicate unimpeded gene flow between the two forms where their breeding ranges meet or overlap. Thus *dealbatus* may be at a phenotypically well-differentiated end of a plumage cline along the East Asian coastline, or there may be a relatively wide zone of intergradation between the two taxa in central eastern China. Studies of breeding populations to the north of Fujian Province are required to assess if there is any clinality or intermediacy of phenotypic traits. Based on the genetic evidence, we do not support calls for the elevation of *dealbatus* to biological species level. However, on account of phenotypic differences *dealbatus* should continue to be considered a subspecies of the Kentish Plover (*C. alexandrinus*).

### Potential mechanisms of the genetic-phenotypic disagreement

Little is known about the underlying mechanisms that may account for a conflict between phenotypic and genotypic differentiation as seen in *dealbatus* and *alexandrinus*. It has been shown that speciation processes can be mediated by a select number of key genes, so called ‘genomic islands of speciation’, in the absence of any notable neutral genomic differentiation [50], [51]. In birds, this has been confirmed in monarch flycatchers where radically different plumages are based on a single mutation [52].

On the other hand, new research into changes in gene expression (e.g. [53]) suggests that sequence data may contain limited information on the origin of important phenotypic differences between sister taxa. Key traits such as wing patches in flies [54] or beak size in Darwin's finches [55] are confirmed to be subject to regulatory variation modulating gene expression. Similarly, Carrion and Hooded Crows (*Corvus corone corone* and *C. c. cornix*, respectively) are phenotypically well-differentiated (i.e. all black versus black-and-grey) but display limited genetic differentiation at 25 neutral nuclear introns [12]. However, there is notable differentiation in 1,300 genes expressed in the crows' brains.

The most likely conclusion of our data is that the White-faced Plover is probably a young lineage whose phenotypic traits are encoded by a limited number of genes, whereas few additional genomic differences have so far accumulated. Its diagnostic plumage traits may additionally be governed by differences in gene expression that would be undetectable by sequence analysis. Future research on *dealbatus* should (1) focus on candidate loci for plumage pigmentation (e.g. [52]) and (2) incorporate gene expression scans, since expression divergence may evolve faster than nucleotide divergence, possibly due to correlated effects that the change of expression of one gene has on other genes (e.g [12]).

### Phylogenetics of the *C. alexandrinus* superspecies

Our results provide the first glimpse into the evolutionary history of the Kentish Plover superspecies and establish the phylogenetic relationships of five members of this species complex with firm nodal support (Fig. 3). In particular, we confirm that *C. nivosus* and *C. alexandrinus* are not conspecific [18], and that south-east Asian *C. peronii* – which is not always considered a member of this superspecies (e.g. [19]) – is actually embedded in the complex and may constitute the sister lineage of African *C. marginatus* pending further sampling. Our data point to unusual levels of intra-specific differentiation between Malagasy and African populations of *C. marginatus*, but more data are needed to re-assess their level of differentiation.

Using both microsatellites and mtDNA genes, we sampled 208 individuals of *C. a. alexandrinus* (not including *dealbatus*) from sites across the whole of Eurasia. We had a large sample size (n>15) for each Spain, Ukraine, Turkey, the Arabian peninsula, and Taiwan (including winter and summer individuals), and a moderate sample size (5<n≤15) for the Indian subcontinent, Japan and north-east China, with additional samples from Xinjiang in central Asia (Fig. 2). *C. alexandrinus* displayed no detectable mtDNA (Fig. 3) or microsatellite (Fig. 4) differentiation across this vast range. This lack of differentiation supports previous findings that continental Eurasian populations of *C. alexandrinus* must be connected by high levels of gene flow [18]. However, more sampling of *C. alexandrinus* is required, especially in marginal and insular localities of its range (e.g. European or North African islands), to assess if this extreme genetic homogeneity extends to all populations.

In conclusion, we show that morphological and genetic differentiation are decoupled between White-faced and Kentish Plovers. In addition, our work reveals novel insights into the distribution of a cosmopolitan superspecies of shorebird that has served as a model organism in evolutionary and ecological research (e.g. [15], [16], [17]). To fully resolve the root of the conflicting morphological and genetic data, future research will benefit from the incorporation of genome-wide sequences, from a focus on candidate loci for plumage pigmentation or from gene expression scans to characterize expression divergence.

## Supporting Information

File S1 Contains the following supporting material: Methods; Table S1. Sample identities and localities; Table S2. Evolutionary models and parameters selected for each locus by jModelTest.
(DOC)

## References

[1] Darwin C (1859). On the origin of species by means of natural selection, or the preservation of favoured races in the struggle for life.

[2] Avise JC (1994). Molecular markers, natural history, and evolution.

[3] Hedges SB, Kumar S (2009). The timetree of life.

[4] Mayr G (2011). The phylogeny of charadriiform birds (shorebirds and allies) - reassessing the conflict between morphology and molecules.. Zoological Journal of the Linnean Society.

[5] Funk DJ, Omland KE (2003). Species-level paraphyly and polyphyly: Frequency, causes, and consequences, with insights from animal mitochondrial DNA.. Annual Review of Ecology Evolution and Systematics.

[6] Olsson U, Alström P, Ericson PGP, Sundberg P (2005). Non-monophyletic taxa and cryptic species - Evidence from a molecular phylogeny of leaf-warblers (*Phylloscopus*, Aves).. Molecular Phylogenetics and Evolution.

[7] Rheindt FE, Norman JA, Christidis L (2008). DNA evidence shows vocalizations to be a better indicator of taxonomic limits than plumage patterns in *Zimmerius* tyrant-flycatchers.. Molecular Phylogenetics and Evolution.

[8] Saitoh T, Alström P, Nishiumi I, Shigeta Y, Williams D (2010). Old divergences in a boreal bird supports long-term survival through the Ice Ages.. BMC Evolutionary Biology.

[9] Grant PR, Grant BR (2008). How and why species multiply: the radiation of Darwin's finches.

[10] Ahn SM, Kim TH, Lee S, Kim D, Ghang H (2009). The first Korean genome sequence and analysis: Full genome sequencing for a socio-ethnic group.. Genome Research.

[11] Tishkoff SA, Reed FA, Friedlaender FR, Ehret C, Ranciaro A (2009). The Genetic Structure and History of Africans and African Americans.. Science.

[12] Wolf JBW, Bayer T, Haubold B, Schilhabel M, Rosenstiel P (2010). Nucleotide divergence vs. gene expression differentiation: comparative transcriptome sequencing in natural isolates from the carrion crow and its hybrid zone with the hooded crow.. Molecular Ecology.

[13] Vonholdt BM, Pollinger JP, Lohmueller KE, Han EJ, Parker HG (2010). Genome-wide SNP and haplotype analyses reveal a rich history underlying dog domestication.. Nature.

[14] Rubin CJ, Zody MC, Eriksson J, Meadows JRS, Sherwood E (2010). Whole-genome resequencing reveals loci under selection during chicken domestication.. Nature.

[15] Blomqvist D, Andersson M, Küpper C, Cuthill IC, Kis J (2002). Genetic similarity between mates and extra-pair parentage in three species of shorebirds.. Nature.

[16] Székely T, Thomas GH, Cuthill IC (2006). Sexual conflict, ecology, and breeding systems in shorebirds.. Bioscience.

[17] Küpper C, Kosztolányi A, Augustin J, Dawson DA, Burke T (2010). Heterozygosity-fitness correlations of conserved microsatellite markers in Kentish plovers *Charadrius alexandrinus*.. Molecular Ecology.

[18] Küpper C, Augustin J, Kosztolányi A, Figuerola J, Burke T (2009). Kentish versus Snowy Plover: Phenotypic and genetic analyses of *Charadrius alexandrinus* reveal divergence of Eurasian and American subspecies.. Auk.

[19] Piersma T, Wiersma P, del Hoyo J, Elliott A, Sargatal J (1996). Family Charadriidae (Plovers).. Handbook of the Birds of the World.

[20] Hart E, Jackson AC (1915). Notes on some waders.. Ibis.

[21] Swinhoe R (1863). Catalogue of the birds of China, with remarks principally on their geographical distribution.. Proceedings of the Zoological Society London.

[22] Swinhoe R (1870). On the plovers of the genus *Ægialites* found in China.. Proceedings of the Zoological Society London.

[23] Kennerley PR, Bakewell DN, Round PD (2008). Rediscovery of a long-lost *Charadrius* plover from South-East Asia.. Forktail.

[24] Tobias JA, Seddon N, Spottiswoode CN, Pilgrim JD, Fishpool LDC (2010). Quantitative criteria for species delimitation.. Ibis.

[25] Brooks TM, Helgen KM (2010). Biodiversity: a standard for species.. Nature.

[26] Lee PLM, Prys-Jones RP (2008). Extracting DNA from museum bird eggs, and whole genome amplification of archive DNA.. Molecular Ecology Resources.

[27] Chesser RT (1999). Molecular systematics of the rhinocryptid genus Pteroptochos.. Condor.

[28] Eberhard JR, Bermingham E (2004). Phylogeny and biogeography of the *Amazona ochrocephala* (Aves: *Psittacidae*) complex.. Auk.

[29] Wenink PW, Baker AJ, Tilanus MGJ (1994). Mitochondrial control region sequences in two shorebird species, the turnstone and the dunlin, and their utility in population genetic studies.. Molecular Biology and Evolution.

[30] Funk WC, Mullins TD, Haig SM (2007). Conservation genetics of snowy plovers (*Charadrius alexandrinus*) in the Western Hemisphere: population genetic structure and delineation of subspecies.. Conservation Genetics.

[31] Küpper C, Horsburgh GJ, Dawson DA, Ffrench-Constant R, Székely T (2007). Characterization of 36 polymorphic microsatellite loci in the Kentish plover (*Charadrius alexandrinus*) including two sex-linked loci and their amplification in four other *Charadrius* species.. Molecular Ecology Notes.

[32] Primmer CR, Møller AP, Ellegren H (1995). Resolving genetic relationships with microsatellite markers: A parentage testing system for the swallow *Hirundo rustica*.. Molecular Ecology.

[33] Posada D (2008). jModelTest: Phylogenetic model averaging.. Molecular Biology and Evolution.

[34] Swofford DL (2000). PAUP*: Phylogenetic Analysis using Parsimony (* and Other Methods).

[35] Ronquist F, Huelsenbeck JP (2003). MrBayes 3: Bayesian phylogenetic inference under mixed models.. Bioinformatics.

[36] Shimodaira H, Hasegawa M (1999). Multiple comparisons of loglikelihoods with applications to phylogenetic inference.. Molecular Biology and Evolution.

[37] Excoffier L, Laval G, Schneider S (2005). Arlequin ver. 3.0: An integrated software package for population genetics data analysis.. Evolutionary Bioinformatics Online.

[38] Huelsenbeck JP, Andolfatto P (2007). Inference of population structure under a Dirichlet process model.. Genetics.

[39] Pritchard JK, Stephens M, Donnelly P (2000). Inference of population structure using multilocus genotype data.. Genetics.

[40] Falush D, Stephens M, Pritchard JK (2003). Inference of population structure using multilocus genotype data: Linked loci and correlated allele frequencies.. Genetics.

[41] Jakobsson M, Rosenberg NA (2007). CLUMPP: a cluster matching and permutation program for dealing with label switching and multimodality in analysis of population structure.. Bioinformatics.

[42] Rosenberg NA (2004). Distruct: a program for the graphical display of population structure.. Molecular Ecology Notes.

[43] Wang ZS, Baker AJ, Hill GE, Edwards SV (2003). Reconciling actual and inferred population histories in the house finch (*Carpodacus mexicanus*) by AFLP analysis.. Evolution.

[44] Küpper C, Burke T, Székely T, Dawson DA (2008). Enhanced cross-species utility of conserved microsatellite markers in shorebirds.. BMC Genomics.

[45] Peters JL, Zhuravlev Y, Fefelov I, Logie A, Omland KE (2007). Nuclear loci and coalescent methods support ancient hybridization as cause of mitochondrial paraphyly between gadwall and falcated duck (Anas spp.).. Evolution.

[46] Alström P, Olsson U, Lei F, Wang HT, Gao W (2008). Phylogeny and classification of the Old World Emberizini (Aves, Passeriformes).. Molecular Phylogenetics and Evolution.

[47] Rheindt FE, Christidis L, Norman JA (2009). Genetic introgression, incomplete lineage sorting and faulty taxonomy create multiple cases of polyphyly in a montane clade of tyrant-flycatchers (*Elaenia*, Tyrannidae).. Zoologica scripta.

[48] Green RE, Krause J, Briggs AW, Maricic T, Stenzel U (2010). A Draft Sequence of the Neandertal Genome.. Science.

[49] Rosenblum EB, Harmon LJ (2011). “Same same but different”: replicated ecological speciation at white sands.. Evolution.

[50] Turner TL, Hahn MW, Nuzhdin SV (2005). Genomic islands of speciation in *Anopheles gambiae*.. PLOS Biology.

[51] Harr B (2006). Genomic islands of differentiation between house mouse subspecies.. Genome Research.

[52] Uy JAC, Moyle RG, Filardi CE, Cheviron ZA (2009). Difference in plumage color used in species recognition between incipient species is linked to a single amino acid substitution in the melanocortin-1 receptor.. American Naturalist.

[53] Tautz D (2000). Evolution of transcriptional regulation.. Current Opinion in Genetics & Development.

[54] Gompel N, Prud'homme B, Wittkopp PJ, Kassner VA, Carroll SB (2005). Chance caught on the wing: cis-regulatory evolution and the origin of pigment patterns in *Drosophila*.. Nature.

[55] Abzhanov A, Kuo WP, Hartmann C, Grant BR, Grant PR (2006). The calmodulin pathway and evolution of elongated beak morphology in Darwin's finches.. Nature.

