# Supplementary material for: Conflict between Genetic and Phenotypic Differentiation: The Evolutionary History of a ‘Lost and Rediscovered’ Shorebird
Source: PLoS One. 2011 Nov 9;6(11):e26995. doi: 10.1371/journal.pone.0026995 (PMC3212520; doi:10.1371/journal.pone.0026995)
Supplement: File S1 — Contains the following supporting material: Methods; Table S1. Sample identities and localities; Table S2. Evolutionary models and parameters selected for each locus by jModelTest. (DOC) [file pone.0026995.s001.doc]

Supplementary Material

**Methods**

*Sampling regime and laboratory techniques*

For DNA obtained from toe pad samples that was partially degraded, we designed primers (all of which had an annealing temperature of 55°C) to amplify shorter fragments. The ND3 fragment was split into two shorter fragments with the primer pairs N01shortF (CCGAAATCAACTGTCTTTGTT) and N01shortR (CTTCCTCAGTAGCAATCCTATTC) as well as N02shortF (AGGATTGCTACTGAGGAAGAA) and N02shortR (CCCAGAGAAGAGCAATCAAC), respectively. The ATPase 6/8 fragment was split into four fragments with the following four primer pairs: (1) A01shortF (ACTTCGAGAGCTGATCCTCA) and A01shortR (GTGGGTTGGTGTGAGTGAGT); (2) A02shortF (AACTATTATCACTCACTCACACCAA) and A02shortR (AGGGTGGAGAGTCGGTTAGT); (3) A03shortF (TACTCCCCGCACCAGATAAT) and A03shortR (GCTAGTGCTATGTTTATTGATAGTTGA); (4) A04shortF (CTCCCCTACACCTTCACTCC) and A04shortR (GTGAGGCGGACTCCTAGTG). The CR fragment was split into three shorter fragments with three primer pairs: (1) C01shortF (CATACACCTGCATGTACTAAGTT) and C01shortR (AGTAGGTACTTAGGCACTGTCG); (2) C02shortF (AATTAATGATACGACAGTGCCTAA) and C02shortR (AGAATGGGCCTGAAGCTAGT); (3) C03shortF (ACACCTCACGTGAAATCAGC) and C03shortR (GAGAACCAAAAGCAGAGAAGC). One sample from Saudi-Arabia (extracted from blood) was analysed using primers for both long and short fragments. The assembled sequences did not differ using the different primer sets and therefore we are confident that we amplified the same target region with primers for long and short fragment combinations.

*Phylogenetic Methods*

We employed the Akaike information criterion as implemented in the program jModelTest (Posada 2008) to assess the best fit for each individual mt gene (Table S2). We utilized maximum parsimony (MP) and Bayesian methods using the programs PAUP* 4.0b10 (Sinauer Associates, Inc.; see also Swofford 2002) and MRBAYES 3.1.2 (Ronquist & Huelsenbeck 2003), respectively.

Heuristic MP searches were run with PAUP’s tree-bisection-reconnection method for tree-swapping by stepwise addition using random addition sequence. MP support for individual nodes was estimated through heuristic bootstrap re-sampling, using 100 replicates for single-gene analyses and 1000 replicates for the concatenated dataset.

For the purposes of using the best-fit model as given by jModelTest in our Bayesian analyses, we specified only the number of substitution types and the basic model properties for among-site rate variation (e.g. gamma-distributed or equal rate variation with or without a proportion of invariable sites). We let MRBAYES estimate the particular parameter values of the evolutionary model (such as base frequencies, the rate matrix and the gamma shape parameter value), since in Bayesian analysis there is a moderate computational penalty associated with estimating parameters as opposed to fixing them prior to analysis (Ronquist & Huelsenbeck 2003). The models of each separate partition were used and all parameters were unlinked among partitions in our MRBAYES analysis of the concatenated dataset. For the two coding loci (ATPase and ND3), sequences were partitioned according to first, second and third codon position, with model parameters allowed to vary. In ND3, we took into account the frame shift that occurs in position 173 of the gene relative to the chicken genome (Mindell *et al.* 1998).

In our MRBAYES searches, we conducted two runs each with four chains (one hot, three cold) for 20,000,000 generations, sampling trees every 1,000 generations to evaluate posterior probabilities. We inspected likelihood vs. generation plots in Tracer Version v1.5 (Rambaut & Drummond 2007) to ascertain how many generations each run required to reach a likelihood plateau. For all three individual loci, each of the two runs per locus reached a likelihood plateau after ~100,000 generations or less, so we excluded the first 400,000 generations (2%) to set a conservative burn-in value. For the concatenated dataset, in contrast, both runs reached a likelihood plateau at ~2,000,000 generations, so we excluded the first 3,000,000 generations (15%) as burn-in. We additionally evaluated convergence of different runs using Tracer, making sure that Bayesian runs reached an effective sample size greater than 200 at the corresponding burn-in. For the control region data, one of the two runs did not reach an effective population size of 200, but discarding that run did not change the tree topology at any of the strongly supported clades, so we retained it (data not shown).

**Table S1** Ingroup samples and their sampling localities; abbreviations: N – number of samples; NHM – Natural History Museum (Tring, UK); NMW – Naturhistorisches Museum Wien (Vienna, Austria).

| Taxon | Sample locality | Type of sample; collection year(s); collector (if needed) | N total | N control region | N ATPase | N ND3 | N microsatellites | Voucher numbers (for museum samples); collector and collecting year (for non-museum samples) |
| --- | --- | --- | --- | --- | --- | --- | --- | --- |
| *dealbatus* | Xiamen, Fujian Province, China | toe pad | 6 | 5 | 3 | 5 | 4 | NHM 1893.1.25.194; NHM 1896.7.1.560; NHM 1896.7.1.562; NHM 1896.7.1.563; NHM 1896.7.1.568; NHM unreg ‘16D’ |
| *dealbatus* | Zhanjiang, Guangdong Province | toe pad | 7 | 7 | 5 | 7 | 6 | NHM 1935.10.23.75, NHM 1935.10.23.76, NHM 1935.10.23.77, NHM 1935.10.23.78, NHM 1935.10.23.79, NHM 1935.10.23.80, NHM 1935.10.23.81 |
| *dealbatus* | Hainan Island, China | toe pad | 1 | 1 | 1 | 1 | 1 | NHM 1896.7.1.574 |
| *alexandrinus* | Japan: Ibaraki, Okinawa and Chiba prefectures | blood; 2004-2009; Y. Shigeta | 10 | 9 | 10 | 10 | 10 | Yoshimitsu Shigeta (2004-2009) |
| *alexandrinus* | Yokohama, Japan | toe pad | 2 | 2 | 0 | 2 | 1 | NHM 1896.7.1.557; NHM 1893.1.25.201 |
| *alexandrinus* (breeding individuals only) | Tongsiao Township, Taiwan | blood | 25 | 10 | 11 | 11 | 25 | Wei-Ting Liu (2005-2007) |
| *alexandrinus* (wintering individuals only) | Tongsiao Township, Taiwan | blood | 22 | 8 | 5 | 9 | 22 | Wei-Ting Liu (2005-2007) |
| *alexandrinus* | Bohai, Tangshan, Hebei, China | blood | 10 | 10 | 10 | 10 | 5 | Wei-Pan Lei (2009) |
| *alexandrinus* | Junggar Basin, Xinjiang Province, China | blood | 7 | 1 | 1 | 1 | 7 | Tamás Székely (2008) |
| *alexandrinus* | Karachi, Sindh, Pakistan | toe pad | 1 | 1 | 0 | 1 | 0 | NHM 1941.5.30.8651 |
| *alexandrinus* | Kotri, Sindh, Pakistan | toe pad | 1 | 1 | 1 | 1 | 0 | NHM 1941.5.30.8655 |
| *alexandrinus* | Jhelum, Punjab, Pakistan | toe pad | 3 | 3 | 2 | 3 | 2 | NHM 1949.Whi.1.1483; NHM 1949.Whi.1.1484; NHM 1949.Whi.1.1485 |
| *alexandrinus* | Patna, Bihar, India | toe pad | 3 | 2 | 2 | 3 | 2 | NHM 1949.Whi.1.1492; NHM 1949.Whi.1.1490; NHM 1949.Whi.1.1491 |
| *alexandrinus* | Farasan Islands, Jizan, Saudi Arabia | blood | 1 | 1 | 1 | 1 | 0 | Monif AlRashidi (2008) |
| *alexandrinus* | Sharjah Creek, Dubai, United Arab Emirates | toe pad | 1 | 1 | 1 | 1 | 1 | NHM 1954.53.6 |
| *alexandrinus* | AlWathba, Abu Dhabi, United Arab Emirates | blood | 25 | 16 | 15 | 17 | 25 | András Kosztolányi & Clemens Küpper (2005-2006) |
| *alexandrinus* | Kuyalnik, Odessa, Ukraine | blood | 17 | 17 | 17 | 17 | 15 | Tamás Székely (2006) |
| *alexandrinus* | Coto de Doñana, Andalusia, Spain | blood | 25 | 17 | 17 | 16 | 25 | András Kosztolányi (2004) |
| *alexandrinus* | Coto Doñana, Andalusia, Spain | toe pad | 1 | 0 | 0 | 1 | 0 | NHM 1934.1.1.2177 |
| *alexandrinus* | Tuzla, Adana Province, Turkey | blood | 25 | 17 | 15 | 16 | 25 | Clemens Küpper (2004) |
| *nivosus* | Ceuta, Sinaloa, Mexico | blood | 25 | 15 | 14 | 13 | 25 | Clemens Küpper (2006) |
| *marginatus* | Port Nolloth, Northern Cape Province, South Africa | toe pad | 2 | 1 | 2 | 2 | 0 | NHM 1903.10.14.282a; NHM 1903.10.14.280 |
| *marginatus* | unspecified localities on Madagascar west coast | blood | 25 | 13 | 12 | 8 | 25 | Sama Zefania (2002-2005) |
| *peronii* | unspecified locality in Borneo | toe pad | 1 | 1 | 1 | 1 | 0 | NMW 64.564 |
| *ruficapillus* | West Bastion, Wyndham, Western Australia | toe pad | 1 | 1 | 1 | 1 | 0 | NHM 1969.4.54 |
| *ruficapillus* | Oyster Harbour, Albany, Western Australia | toe pad | 1 | 1 | 1 | 1 | 0 | NHM 1905.12.26.497 |
| *ruficapillus* | Gippsland, Victoria, Australia | toe pad | 1 | 1 | 1 | 1 | 0 | NHM 96.7.1.606 |
| *ruficapillus* | Clifton Hills, South Australia, Australia | toe pad | 2 | 2 | 1 | 1 | 0 | NHM 1965.43.17; NHM 1965.43.16 |
| *ruficapillus* | Cheetham Wetlands, Melbourne, Victoria, Australia | blood | 25 | 15 | 15 | 16 | 25 | Mike Weston & Clemens Küpper (2010) |
| *ruficapillus* | unspecified locality in Australia | toe pad | 2 | 2 | 2 | 2 | 0 | NMW 49.121; NMW 49.122 |

**Table S2** Evolutionary models and parameters selected for each locus by jModelTest.

| Model parameters | ATPase | Control region | ND3 |
| --- | --- | --- | --- |
| Model type | TIM3+I+G | TIM2+G | HKY+I |
| Base frequencies (A, C, G, T) | 0.2395, 0.0868, 0.3649, 0.3087 | 0.2744, 0.1495, 0.3075, 0.2685 | 0.2691, 0.1263, 0.2932, 0.3115 |
| Rate matrix (AC, AG, AT, CG, CT, GT) | 4.4377, 23.2696, 1, 4.4377, 164.9713, 1 | 0.5395, 21.0429, 0.5395, 1, 14.9481, 1 | n/a |
| Gamma shape | 0.984 | 0.113 | equal rates (no gamma) |
| Number of categories to divide discrete approximation of gamma distribution | 4 | 4 | n/a |
| Transition / transversion ratio | n/a | n/a | 26.1919 |
| Proportion of invariable sites | 0.78 | 0 | 0.861 |
